# Construction of infectious clones of tomato torrado virus and their delivery by agroinfiltration

**DOI:** 10.1007/s00705-014-2266-1

**Published:** 2014-11-23

**Authors:** Przemysław Wieczorek, Marta Budziszewska, Aleksandra Obrępalska-Stęplowska

**Affiliations:** Interdepartmental Laboratory of Molecular Biology, Institute of Plant Protection, National Research Institute, Władysława Węgorka 20 St, 60-318 Poznań, Poland

## Abstract

The first biologically active infectious clones of tomato torrado virus (ToTV) were generated and delivered into *Nicotiana benthamiana* and *Solanum lycopersicum* plants *via* *Agrobacterium tumefaciens*. The engineered constructs consisted of PCR-amplified complementary DNAs derived from the ToTV RNA1 and RNA2 components, individually inserted into an engineered pGreen binary vector between the CaMV 35S promoter and nopaline synthase terminator. These constructs were introduced into the plant hosts by means of *A. tumefaciens*-mediated infiltration. In the presence of the progeny virus, typical symptoms of ToTV infection developed in *N. benthamiana* and *S. lycopersicum*. Moreover, the virus was sap-transmissible when isolated from agroinfiltrated plants and induced symptoms similar to those caused by the wild-type virus. The presence of viral particles and viral genetic material was confirmed by electron microscopy and re-inoculation to *S. lycopersicum* and *N. benthamiana,* as well as by reverse transcription polymerase chain reaction and high-resolution melt analysis.

**Electronic supplementary material:**

The online version of this article (doi:10.1007/s00705-014-2266-1) contains supplementary material, which is available to authorized users.

Tomato torrado virus (ToTV) is the type member of the genus *Torradovirus* in the family *Secoviridae* [1]. This viral pathogen is the causal agent of “torrado disease”, which is characterized by burn-like severe systemic necrosis of leaves, stems, and fruits of infected tomato plants; these symptoms dramatically decrease the consumer value of the tomato crop. Like other members of the genus *Torradovirus*, ToTV contains a bipartite single-stranded RNA genome (RNA1 is ca. 7800 nt and RNA2 is ca. 5400 nt, both polyadenylated) and forms icosahedral particles [2]. The viral RNAs serve as templates for translation of polyproteins that are processed into mature nonstructural (RNA1 and RNA2) and structural proteins (RNA2). The virus is transmitted by *Bemisia tabaci* [2] and *Trialeurodes vaporariorum* [3]; however, under experimental conditions it can be transmitted mechanically. In 2008, the first complete sequence of a Polish isolate of ToTV-Wal′03 was described [4], whereas characterization of a second Polish isolate, ToTV-Kra, in 2014 revealed intra-isolate genetic variability in the 3′ untranslated region (3′UTR) of RNA1 [5]. Several detection methods were developed to identify ToTV and other torradoviruses in infected crops [6, 7].

In order to conduct more-precise studies of ToTV gene functions, it was essential to generate ToTV infectious clones to use them in further functional studies. Infectious clones of plant RNA viruses are basic tools in molecular virology. The cloned viral genomes can be easily modified, which allows researchers to conduct genetic studies and determine viral gene functions. Infectious clones can also be used to study virus–vector–plant interactions. To date, infectious DNA clones (or infectious transcripts) of a number of plant viruses have been described and utilized in basic research [8–11]. These infectious clones have been used to characterize pathogenicity determinants of viruses and to determine viral gene function in host cells [8, 12, 13], to express heterologous proteins in plants [14, 15], or as virus-induced gene silencing platforms [16, 17].

Here, we describe the generation of infectious clones of a Polish isolate of ToTV. This was accomplished by i) reverse transcription (RT) polymerase chain reaction (PCR)-based complementary DNA (cDNA) amplification of complete sequences of ToTV RNA1 and RNA2, ii) recombination-based cloning of the amplified cDNA into a binary vector, and iii) *Agrobacterium*-mediated *in vivo* transcription of viral RNAs in inoculated plants. To our knowledge, these are the first biologically active infectious clones of ToTV or any member of the genus *Torradovirus*.

Virus isolates (ToTV Kra_2014; accession numbers of RNA1 and RNA2, KJ940975 and KJ940974, respectively) were maintained in *Nicotiana benthamiana* and *Solanum lycopersicum* plants in the greenhouse with a photoperiod and temperature of 16 h-28 °C/8 h-24 °C (day/night). Total RNA was isolated from systemic leaves collected from symptomatic ToTV-infected *N. benthamiana* plants using TRI Reagent and used for RT with Superscript III Reverse Transcriptase (Life Technolgies) and primer asTo2C_RV (sequences of primers used in the study are listed in Supplementary Table S1). The resulting cDNA was used in PCR with *PfuUltra* High-Fidelity DNA Polymerase (Agilent Technologies), and the asTo1A_FW/asTo2C_RV and asTo2A_FW/asTo2C_RV primers were used for amplification the full-length cDNAs of RNA1 and RNA2, respectively. Each cDNA molecule was flanked at the 5′ and 3′ termini by a short sequence providing the required homology to the 5′ and 3′ termini of the linearized binary vector used for cloning of the cDNA molecules. The cloning workflow is described in the Supplementary Fig. S1.) A backbone cloning vector (pgR107), derived from the pGreen plasmid, was amplified by means of inverse PCR, using the pgR107 vector (kindly provided by Prof. D. Baulcombe) [18] as a template and the primers pgR107de_FW/pgR107de_1RV or pgR107de_FW/pgR107de_2RV (indicated in Supplementary Fig. S1) and *PfuUltra* High-Fidelity DNA Polymerase (Agilent Technologies). After the PCR, the remaining original plasmid template in the mixture was digested using the restriction enzyme *DpnI* (Thermo Scientific). The resulted linearized vectors, namely pGreenMod1 and pGreenMod2, were flanked at both 5′ and 3′ termini by short sequences corresponding to the 5′ and 3′ termini of the ToTV-derived cDNAs. These sequences provided the essential intra-molecular sequence homology needed for recombination.

Assembly of the cDNA1- and cDNA2-derived PCR products with corresponding cloning vectors was done by means of homologous recombination. Briefly, 50 ng of the linearized vector (pGreenMod1 or pGreenMod2) was mixed with 200 ng of the corresponding PCR product (originating from RNA1 or RNA2) and GeneArt^®^ 2X Enzyme Mix (Life Technologies). The reaction mixture was incubated for 30 min at room temperature and then used directly for transformation of *E. coli* One Shot^®^ DH10B™ T1R SA Cells (Life Technologies). From initially PCR-verified transformants, plasmid DNAs were isolated and sequenced.

We made it a priority that the first nucleotide of each of the ToTV RNA1 and RNA2 cDNAs (inserted in pGreenMod vectors) was precisely transcribed from the cauliflower mosaic virus (CaMV) 35S promoter (Fig. 1), thereby avoiding the introduction of any non-viral nucleotides during *in vivo* transcription. The 3′ untranslated region (UTR) was followed by a tract of 25 adenine residues and the NOS terminator. It has been shown that extra non-viral nucleotides added to the 5′ terminus of genomic RNA transcripts might significantly reduce virus infectivity [19, 20]. This occurs when the RNA transcription is driven from the T7 RNA polymerase promoter, because, for optimal activity, the promoter needs at least one additional guanine residue, which in turn is transcribed as the first ribonucleotide in the resulting RNA transcript). This problem can be avoided by introducing the template to be transcribed downstream of the CaMV 35S promoter. Precise fusion of the CaMV 35S promoter with a DNA can be done by means of the overlap-extension PCR method as described previously [21]. However, this approach still needs to be followed by additional manipulations—for example a long-range PCR [21]—but this might be accompanied by errors introduced into long, engineered DNA molecules. Therefore, the recombination-based cloning of the long DNAs, as described in the study, was chosen and performed instead of blunt-end ligation and other time-consuming cloning strategies.

**Fig. 1:**
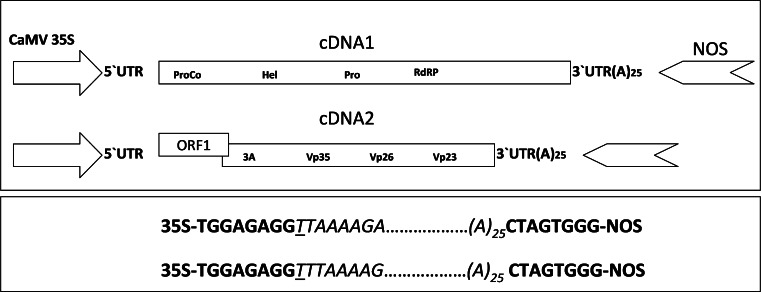
Schematic diagram of DNA constructs representing the expression cassettes inserted into the pGreen vector used as a backbone for the *in vivo* reconstitution of RNA1 and RNA2 of p35ToTV-Kra (upper panel). The detailed CaMV35S/cDNA/NOS junction site formed after recombination of pGreenMod1 and pGreenMod2 with ToTV-Kra cDNA1 and cDNA2, respectively, is indicated. The underlined nucleotide indicates the first transcribed nucleotide (lower panel). ProCo, protease cofactor; Hel, helicase; Pro, protease; RdRP, RNA-dependent RNA polymerase; ORF1, protein of unknown function; 3A, movement protein; Vp35, Vp26, Vp23, subunits of capsid and UTR, untranslated region

The resulting engineered recombinant plasmids, named p35Kra1 and p35Kra2, which together constitute the p35ToTV-Kra genome, were each introduced by transformation into *A. tumefaciens* GV3101 (harboring pSoup helper plasmid to enable replication of the pGreen-based p35Kra1 and p35Kra2 plasmids in the *Agrobacterium* cells). After 48 h of growth at 28 °C on Luria-Bertani (LB) medium supplemented with kanamycin (50 μg/ml) and tetracycline (5 μg/ml), five random *A. tumefaciens* colonies were picked and grown individually in liquid LB medium (supplemented with above-mentioned antibiotics) for another 48 h at 28 °C. The bacterial cells were centrifuged and resuspended in infiltrating medium (10 mM MgCl_2_, 0.5 μM acetosyringone, 10 mM MES, pH 5.8) to an OD_600nm_ of 1.0 and incubated at room temperature for another 4 h. Equal amounts of p35Kra1- and p35Kra2-transformed *A. tumefaciens* suspensions were mixed together prior to infiltration of 4-week-old seedlings of *N. benthamiana* and *S. lycopersicum* (var. Beta Lux). The agroinfiltration was done using two leaves of five seedlings of each host. Simultaneously, *N. benthamiana* and *S. lycopersicum* were mechanically inoculated with sap prepared from ToTV-infected tomato and *N. benthamiana* plants. The agroinfiltrated and mechanically inoculated plants were maintained in a greenhouse with a photoperiod and temperature of 16 h-28 °C/8 h-24 °C (day/night). All of the tested plants were spatially isolated within the chamber and were kept there in separate insect-rearing cages free from insects. Mock-inoculated plants were used as controls to exclude any unintended contamination. The experiments were performed in triplicate.

In nature, ToTV-Kra causes characteristic torradovirus symptoms, including yellowing and ToTV-specific spoon-like malformations of systemically infected leaves in *N. benthamiama* and leaf mottling followed by severe necrosis developing near veins of systemically infected leaves in *S. lycopersicum* (Fig. 2) [2]. Similar symptoms appeared in *N. benthamiana* (ca. 100 % of analysed plants) and *S. lycopersicum* (ca. 95 % of analysed plants) agroinfiltrated with p35ToTV-Kra 13 and 17 days after infiltration, respectively (Fig. 2). In contrast, in sap transmission assays, only ca. 80 % and ca. 90 % of mechanically inoculated tomato and *N. benthamiana*, respectively, developed ToTV-specific symptoms.

**Fig. 2:**
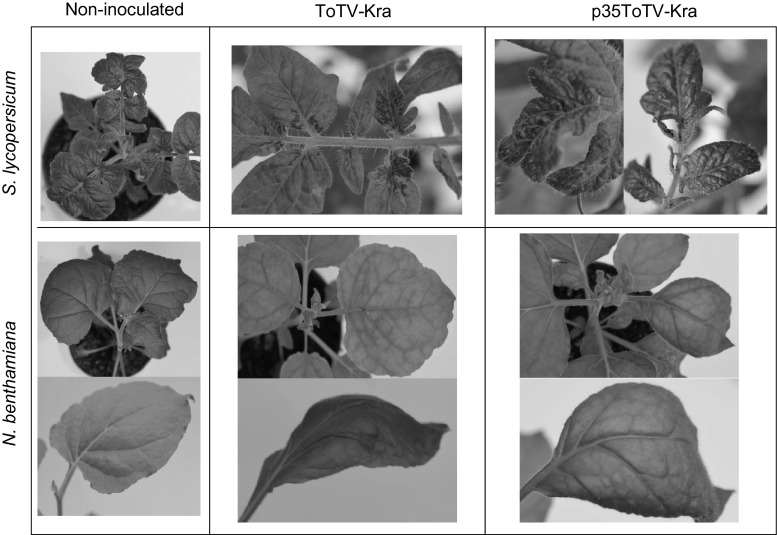
Comparison of symptoms induced by ToTV-Kra (center panel) and p35ToTV-Kra (right panel) on *N. benthamiana* (13 days post inoculation) and *S. lycopersicum* var. Beta Lux (17 days after inoculation). Healthy plants are shown on the left

To initially confirm the presence of torradovirus virions in the infected plants, leaf-dip preparations of symptomatic *N. benthamiana* followed by electron microscopic analysis were performed. The analysis of p35ToTV-Kra infected plants revealed the presence of ca. 28-nm-diameter icosahedral particles, similar to those identified in plants infected with the wild-type virus (Supplementary Fig. S2). Importantly, very few visible particles were identified within the areas observed with the electron microscope. This was noticed in leaf-dip preparations from both p35ToTV-Kra and wild-type ToTV-infected plant material (Supplementary Fig. S2). However, as described previously [2, 22, 23], one of the characteristic features of ToTV is the formation of compact crystalline aggregates, and non-aggregated virus particles are infrequently observed in infected tissues.

To confirm the infectivity of the virions generated in symptomatic plants following inoculation with p35ToTV-Kra, infected plant material was collected and used for mechanical inoculation of *N. benthamiana* and *S. lycopersicum* (var. Beta Lux) seedlings. The first symptoms of p35ToTV-Kra infection observed on inoculated plants appeared 15-17 days after inoculation and were similar to those observed for wild-type virus (data not shown). Moreover, to verify the presence of ToTV genomic viral RNAs in systemic leaves of p35ToTV-Kra-infected plants, RT-PCR was performed. cDNA was prepared on a template of total RNA extracted from upper leaves taken from infected *N. benthamiana* and *S. lycopersicum*. The cDNA was subsequently used for duplex PCR with primers mxToT1a/mxToT1b, which anneal with the RNA-dependent RNA polymerase gene (RNA1), and primers mxToT2a/mxToT2b, which anneal with the one of the coat protein genes (RNA2) of ToTV. Agarose gel electrophoresis of the PCR-amplified DNA fragments from the positive control revealed ToTV-specific bands of the two expected sizes, 289 base pairs (bp) and 470 bp, which correspond to the amplified fragments from RNA1 and RNA2, respectively. Two DNA bands of the expected size were also identified after PCR was done with the cDNA template prepared from *N. benthamiana* and *S. lycopersicum* plants that were infected following infiltration with p35ToTV-Kra (Fig. 3, lanes 1-8). The same RT-PCR products were amplified from RNA isolated from plants infected with the wild-type virus (Fig. 3, lanes 9-10). The presence of ToTV-specific bands amplified after RT-PCR from RNA isolated from p35ToTV-Kra-infiltrated plants was also confirmed by high-resolution melt (HRM) analysis (Supplementary Fig. S3). The analysis revealed that similar HRM dissociation plot patterns were obtained for RT-PCR products from plants infected after mechanical inoculation with the wild-type virus and agroinfiltration with p35ToTV-Kra. In contrast, the two ToTV-specific bands (or HRM peaks) were not detected in the PCR performed with cDNA prepared from RNA isolated from mock-inoculated plants, and therefore, any unintended contamination can be excluded.

**Fig. 3:**
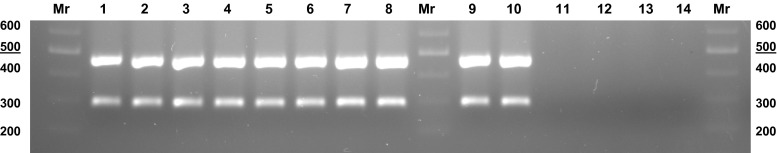
RT-PCR detection of p35ToTV-Kra RNA1 (the lower band) and RNA2 (the upper band). DNA fragments were amplified from total RNA extracted from leaves of *S. lycopersicum* (1-4) and *N. benthamiana* (5-8) plants that were systemically infected with p35ToTV-Kra. Similar results were also obtained for tomato (9) and *N. benthamiana* (10) plants infected with wild-type ToTV-Kra. Healthy plants (11-12) as well as non-template controls (13-14) are also indicated. Mr, DNA size markers

Taken together, we describe here the establishment of infectious RNA1 and RNA2 clones of ToTV and their delivery into *N. benthamiana* and tomato plants, where they resulted in the development of typical torradovirus disease symptoms. The virus generated from the infectious clones infects *N. benthamiana* and *S. lycopersicum* efficiently and induces disease symptoms similar to those that are characteristic of wild-type virus. The progeny virus was sap-transmissible, as plants mechanically inoculated with sap induced symptoms similar to those observed after agroinfiltration of p35ToTV-Kra. Therefore, the ToTV agroinoculation system can be used for genetic manipulations of the ToTV genome and to study virus-encoded virulence determinants as well as viral factors directly involved in virus-vector-plant interactions.

## Electronic supplementary material

Below is the link to the electronic supplementary material.

Supplementary material 1 (DOC 172 kb)
