# Supplementary material for: Construction of infectious clones of tomato torrado virus and their delivery by agroinfiltration
Source: Arch Virol. 2014 Nov 23;160(2):517–21. doi: 10.1007/s00705-014-2266-1 (PMC4315485; doi:10.1007/s00705-014-2266-1)
Supplement: Supplementary file 1 — Supplementary material 1 (DOC 172 kb) [file 705_2014_2266_MOESM1_ESM.doc]

**Supplements:**

**Supplementary Figure S1** Schematic representation of recombination-based assembly of p35ToTV-Kra infectious clones. As an example, the cloning of the RNA1 of ToTV is shown.

The described cloning procedure consists of three stages: A, PCR-based amplification of ToTV RNA1, B, linearization of a binary vector, C, *in vitro* recombination-based assembly of the p35ToTV1 from the amplified ToTV-derived PCR template and vector.

**A1.** Design of the ToTV-specific primers. The ToTV-specific part of each primer (solid line) was followed by 8 nucleotides specific to the cauliflower mosaic virus (CaMV) 35S promoter (forward primer, dotted line) or nopaline synthase (NOS) terminator (reverse primer, dotted line). This provides the 15-bp homology essential for DNA recombination. **A2.** The primers annealed to the 5´ (asTo1A_FW) and 3´ (asTo2C_RV) termini of ToTV RNA1. The black line represents sequences of untranslated regions (UTR) of RNA1, whereas the grey box indicates the ToTV polyprotein coding sequence. **A3.** After PCR, the resulting product represents a full-length cDNA copy of RNA1 flanked by 8-bp sequences of the CaMV 35S promoter and the NOS terminator at the 5´ and the 3´ termini of the amplified DNA (external boxes with vertical lines), respectively.

**B1**. Primers used to prepare the linearized binary vector. The 3´ termini of each primer (dotted line) annealed to template DNA of the chosen vector. The vector-specific region of the primers is followed by 8 nucleotides (solid line) from the 5´ (reverse primer) and the 3´ (forward primer) termini of RNA1 of ToTV. This provides the 15-bp homology essential for DNA recombination. **B2**. The primers annealed to the CaMV 35S promoter sequence (pgR107de_1RV, box with vertical lines) and the NOS terminator (pgR107de_FW, box with lines pattern). **B3**. After inverse PCR, the estimated sequence of a vector was amplified excluding the sequences between the CaMV 35S and NOS (here: the sequence of potato virus X (PVX), grey box). The obtained vector is flanked by an 8-bp sequence of ToTV RNA1 (black boxes).

**C1**. The PCR-amplified full-length cDNA copy of RNA1 and linearized binary vector were mixed. Recombination events between the PCR product and the vector occured within the 15-bp terminal homologous sequences of the molecules. **C2**. The assembled p35ToTV1 construct is ready for transformation of *Escherichia coli* competent cells.


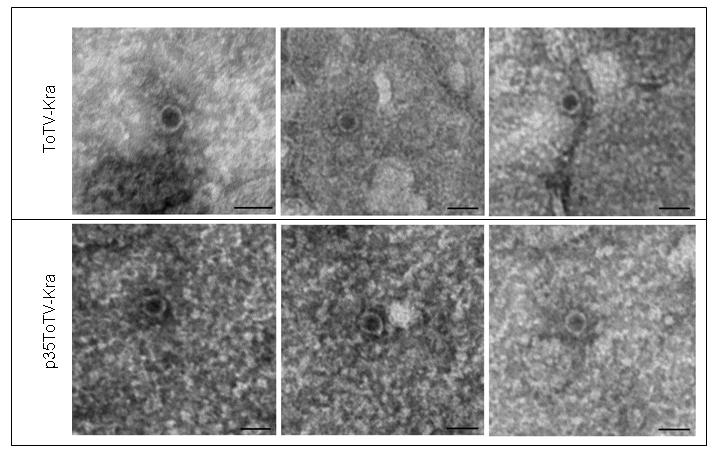


**Supplementary Figure S2**. Electron micrographs showing icosahedral particles identified in leaf-dip preparations obtained from systemically infected leaves of *N. benthamiana* plants infected with the wild-type ToTV-Kra (upper panels) and p35ToTV-Kra (lower panels). The scale bars represent 40 nm.

**Supplementary Figure S3.** High-resolution melt analysis of duplex RT-PCR products with intercalated EvaGreen dye. Two major fluorescence peaks at 85°C and 87.5°C indicate amplified products originating from RNA1 and RNA2, respectively, of ToTV. The black line corresponds to results obtained for the positive control (ToTV-Kra), whereas orange and red plots were generated using samples from plants infected with p35ToTV-Kra.

**Supplementary Table S1.** Primers used in the study.

Primers were designed for the following: amplification of full-length cDNA1 (asTo1A_FW/ asTo2C_RV) and cDNA2 (asTo2A_FW/ asTo2C_RV) of ToTV-Kra; amplification of pGreenMod vector (pgR107de_FW, pgR107de_FW, pgR107de_1RV); multiplex RT-PCR (mxToT1a, mxToT1b, mxToT2a, mxToT2b). ToTV-specific nucleotides are italicized, vector-specific sequences are underlined, and flanking homologous sequences are shown in bold.

| Primer ID | Sequence 5´🡪3´ |
| --- | --- |
| asTo1A_FW | **TGGAGAGG***TTAAAAGAGTTATTTTGAGAATATAAC* |
| asTo2A_FW | **TGGAGAGG***TTTAAAAGAATAATTTTATACAATATTTATGT* |
| asTo2C_RV | **CCCACTAG***TTTTTTTTTTTTTTTTTTTTTTTTTAAAAT* |
| pgR107de_FW | **AAAAAAAA**CTAGTGGGTACCGCG |
| pgR107de_1RV | **TCTTTTAA**CCTCTCCAAATGAAATGAA |
| pgR107de_2RV | **CTTTTAAA**CCTCTCCAAATGAAATGAA |
| mxToT1a | CTCTGCCATGCAAGGTGGGCAT |
| mxToT1b | TCCTGAAGGCATACCTCCCACCA |
| mxToT2a | TACAACAACCAGCCTGCGTGGC |
| mxToT2b | TAGCACTAGCCAAGGGGTGCGT |
